# Response Detection of Castrate-Resistant Prostate Cancer to Clinically Utilised and Novel Treatments by Monitoring Phospholipid Metabolism

**DOI:** 10.1155/2017/4793465

**Published:** 2017-06-22

**Authors:** Tim A. D. Smith, Su M. Phyu, Kholoud S. Alzyoud, Chih-Chung Tseng

**Affiliations:** School of Medicine, Medical Sciences and Nutrition, University of Aberdeen, Foresterhill, Aberdeen AB25 2ZD, UK

## Abstract

Androgen receptor (AR) activation is the primary driving factor in prostate cancer which is initially responsive to castration but then becomes resistant (castration-resistant prostate cancer (CRPC)). CRPC cells still retain the functioning AR which can be targeted by other therapies. A recent promising development is the use of inhibitors (Epi-1) of protein-protein interaction to inhibit AR-activated signalling. Translating novel therapies into the clinic requires sensitive early response indicators. Here potential response markers are explored. Growth inhibition of prostate cancer cells with flutamide, paclitaxel, and Epi-1 was measured using the MTT assay. To simulate choline-PET scans, pulse-chase experiments were carried out with [^3^H-methyl]choline and proportion of phosphorylated activity was determined after treatment with growth inhibitory concentrations of each drug. Extracts from treated cells were also subject to ^31^P-NMR spectroscopy. Cells treated with flutamide demonstrated decreased [^3^H-methyl]choline phosphorylation, whilst the proportion of phosphorylated [^3^H-methyl]choline that was present in the lipid fraction was increased in Epi-1-treated cells. Phospholipid breakdown products, glycerophosphorylcholine and glycerophosphoethanolamine levels, were shown by 31P-NMR spectroscopy to be decreased to undetectable levels in cells treated with Epi-1. LNCaP cells responding to treatment with novel protein-protein interaction inhibitors suggest that ^31^P-NMR spectroscopy may be useful in detecting response to this promising therapy.

## 1. Introduction

Prostate cancer is the second most commonly diagnosed cancer and the 6th leading cause of death in men worldwide [1]. Most prostate cancers are initially sensitive to antiandrogen treatment and advanced stage cases of prostate cancer are treated by antiandrogen therapy, for example, by castration, but such cancers later develop resistance (castrate-resistant prostate cancer (CRPC)) although they are still dependent on the AR (androgen receptor) for growth. The taxane docetaxel has demonstrated some improvement in survival of patients with CRPC and is recommended for patients with CRPC [2].

The development of AR-targeting compounds that can inhibit the growth of CRPC cells is a major step forward in the treatment of this disease. Protein-protein interaction inhibitors are a rapidly emerging class of drugs in anticancer drug development which demonstrate effective therapeutic efficacy towards tumours refractory to other treatments. Work has demonstrated that targeting the androgen receptor with the protein-protein inhibitor molecule family 3-(4-{2-[4-(3-chloro-2-hydroxypropoxy)phenyl]-2-propanyl}phenoxy)-1,2-propanediol (Epi-1) can halt the growth of CRPC cells by inhibiting androgen receptor mediated intracellular signalling [3].

Anticancer treatments are often associated with detrimental side-effects [4, 5]. Early detection of response status can be used to tailor a patient's treatment facilitating cessation of noneffective treatments. Early response detection can also assist in the clinical translation of novel anticancer agents [6]. Molecular imaging techniques such as PET (positron emission tomography) and MRS (magnetic resonance spectroscopy) are sensitive, noninvasive methods for early response detection [7] and cell based studies have shown that changes in metabolite utilisation are early markers of response [8]. FDG ([^18^F]fluoro-2-deoxy-D-glucose) is an unsuitable tracer for imaging the prostate as it is excreted via the kidney so patient FDG-PET scans inevitably show high activity in the pelvic region which will obscure uptake by the prostate gland. However prostate cancer has a high affinity for the phospholipid metabolite, choline, and consequently can be imaged using [^11^C]choline-PET [9]. Further, AR activation in prostate cancer induces signalling via the calcium/calmodulin dependent kinase kinase 2- (CAMKK2-) AMPK signalling pathway which amongst other effects controls phospholipid metabolism so inhibition of signalling via the AR would be expected to induce early changes in phospholipid metabolism which can be probed noninvasively using 31P-NMR spectroscopy. ^31^P-NMR spectroscopy detects the concentration of several phospholipid metabolites including glycerophosphorylcholine (GPC) and phosphocholine (PC) which in some cells has been shown to be associated with proliferation rate [10].

Here prostate cancer cells untreated and treated with flutamide, paclitaxel, or Epi-1 were subject to pulse-chase measurements with [^3^H-methyl]choline (which is chemically identical to [^11^C]choline) and ^31^P-NMR spectroscopy to assess the modulatory effect of these drugs on phospholipid metabolism.

## 2. Methods

### 2.1. Chemicals and Cells

Androgen receptor positive LNCaP prostate cancer cell lines were obtained from the European Collection of Cell Cultures (ETACC). Cells were grown in Dulbecco's Modified Eagle's Medium supplemented with glutaMAX™ (Gibco UK), 10% charcoal stripped serum, and 10,000 units penicillin/10,000 *µ*g and streptomycin per 100 ml of medium. Cells were supplemented in all experiments with the androgen R1881 (1 *µ*g/ml). [^3^H-methyl] choline (2.96 TBq/mmol) was obtained from American Radiochemical Corporation Inc., USA. All chemicals were obtained from Sigma-Aldrich (Poole, UK) unless otherwise stated.

### 2.2. Determination of Drug Induced Cell Growth Inhibition Using the MTT Assay

The cell growth inhibiting effect of anticancer drugs was initially tested using the MTT assay. LNCaP cells in medium (100 *µ*l) were seeded into 96-well plates (75,000 cells/ml) and incubated for 24 h at 37°C in a CO_2_ incubator and then treated with a range of drug concentrations for 72 h at 37°C. Cell number in each well is then determined using the 3-(4,5-dimethylthiazol-2-yl)-2,5-diphenyl tetrazolium bromide (MTT) dye which is converted to a purple colour by mitochondria in viable cells. The intensity of the purple colour, which is proportional to the cell number, was then determined using a scanning multiwell plate reader (MR5000 Dynatech Laboratories Inc., USA) with the absorbance at 540 nm.

### 2.3. Pulse-Chase [^3^H-methyl]choline

The pulse-chase technique simulates exposure of cancer cells in patients to blood levels of [^11^C]choline. LNCaP cells (10^6^ in 5 ml of medium) were seeded in 25 cm^2^ flasks and allowed to establish for 24 h. The cells were then either left untreated or treated with drugs for 48 h after which medium was removed and replaced with 1 ml of medium containing 37 KBq of [^3^H-methyl] choline. After 15 mins, the cells were rapidly washed 3x with ice-cold phosphate buffered saline (Sigma, Poole, UK), then incubated with nonradioactive medium for 1 h. Medium was collected and radioactivity counted and the cells were trypsinized by addition of 0.35 ml of trypsin (0.05% trypsin/EDTA). After the cells were detached, 0.35 ml of medium was added and the cells transferred to 1.5 ml microfuge tubes which were centrifuged at 200*g* for 5 min. The cells were washed by addition of 1 ml of PBS and further centrifugation. The supernatant and medium were combined and subject to phosphate measurement (see below). The cells were subject to fractionation into lipid and aqueous fractions (see below).

### 2.4. Lipid and Aqueous Fractionation

Cells were suspended in 0.375 ml of chloroform : methanol (1 : 2) and left on ice for 1 h with occasional mixing. Separation of the phases was then achieved by addition of 125 *µ*l of 10 mM TRIS buffer and 125 *µ*l of chloroform followed by centrifugation at 10,000*g* for 10 min at 4°C. The upper phase was then removed and subject to phosphate measurement. The lipid phase was removed by carefully accessing with a pipette tip through the cell residue at interface. Radioactivity in the lipid phase was measured by scintillation counting. The cell residue (mainly protein) was dried in the microfuge tubes and then dissolved in 100 *µ*l of NaOH (1 M). A protein assay (Bicinchoninic Acid Protein Assay Kit, Sigma, UK) was carried out on the dissolved pellet after neutralisation with HCl and used to normalise the data for differences in cell number between treated and untreated cells.

### 2.5. Determination of Proportion of Phosphorylated [^3^H-methyl]choline

Aqueous extracts and cell washes were subject to measurement of phosphorylated [^3^H-methyl]choline [11, 12]. Briefly, the total radioactivity in each of the sample was measured using part of each sample and correcting for volume. Phosphates (which included any phosphorylated [^3^H-methyl]choline) were precipitated by addition of equal volumes of Ba(OH)_2_ (0.3 M) and ZnSO_4_ (5% w/v) and centrifugation at 10,000*g* for 10 min to remove the precipitated phosphates. The nonphosphorylated [^3^H-methyl]choline was then determined in the supernatant. The proportion of phosphorylated [^3^H-methyl]choline was determined by subtraction after correcting for dilution [13] from total. The total and phosphorylated activity (including the lipid fraction) were summated and the proportion of phosphorylated activity was determined.

### 2.6. Sample Preparation for ^31^P-NMR Spectroscopy

Cells were seeded in 80 cm^2^ tissue culture flasks and allowed to establish for 24 h. The cells were then either left untreated or treated with drugs for 48 h after which medium was removed and the cells washed 3x with 10 ml PBS. The cells were then detached with trypsin (3 ml) and, after addition of medium (3 ml), the cells were centrifuged at 200*g* for 5 min. The pellet was suspended in isotonic saline and transferred to a 1.5 ml microfuge tube and washed 3x with ice-cold isotonic saline, each time centrifuging at 200*g* for 5 min at 4°C. The pellet was then subject to lipid and aqueous fractionation as described above except 10 mM EDTA and a standard consisting of 1-aminopropylphosphoric acid (0.3 *µ*moles) was added to the initial 0.375 ml of chloroform and methanol. After extraction and phase separation, the aqueous phase was collected and made up to a volume of 0.6 ml by addition of D_2_O. The lipid fraction was spiked with the standard tetramethylsilane (TSP) (0.6 *µ*moles) and subject to ^1^H NMR analysis.

### 2.7. ^31^P-NMR and ^1^H-NMR Spectroscopy

NMR analysis was carried out on a Bruker AVANCE III 400 MHz NMR spectrometer operating at 161.98 MHz for ^31^P with broadband ^1^H decoupling and run overnight (to acquire 20,000 scans) at 25°C. The parameters for each acquisition were as follows: acquisition time 1.258 s; relaxation delay 1 s (peak sizes were similar when 1 s or 2 s were used suggesting complete relaxation in 1 s); pulse angle 45°. At least three independent experiments were carried out and analysed per treatment. Metabolite concentrations were determined by comparison with the standard (0.3 *µ*moles) resonating at 11.8 ppm.

1H-NMR spectra, acquired on the lipid extracts, were run for 200 scans with a relaxation delay of 5 s and a 90° pulse angle.

### 2.8. Statistical Analysis

Significant differences between means were tested using Student's *t*-test. All experiments were carried out at least 3 times.

## 3. Results and Discussion

**Figure 1:**
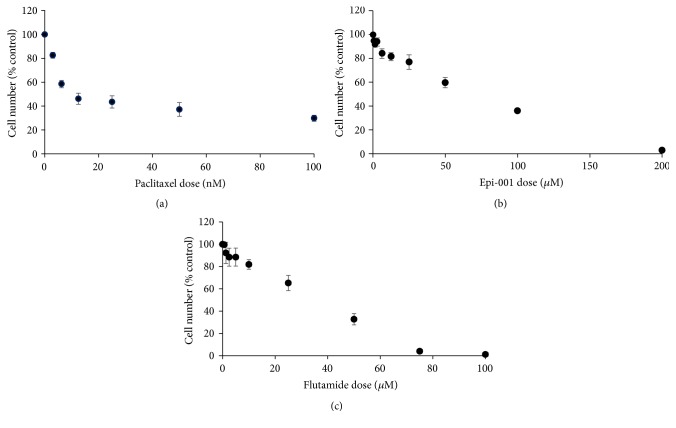
Growth of LNCaP cells treated with paclitaxel (taxol) (a), flutamide (b), or Epi-1 (c) relative to untreated cells.

Figure 1 shows the effect of paclitaxel, Epi-1, and flutamide on the growth of LNCap cells. Growth inhibition of LNCaP cells with paclitaxel achieves a plateau at about 10 nM. A similar effect has been noted previously [14] with this drug. Both Epi-1 and flutamide inhibited growth in a concentration-dependent manner. For subsequent experiments, doses were chosen that decreased growth by about 30% 5 nM paclitaxel, 40 *µ*M Epi-1, and 20 *µ*M flutamide.

**Figure 2:**
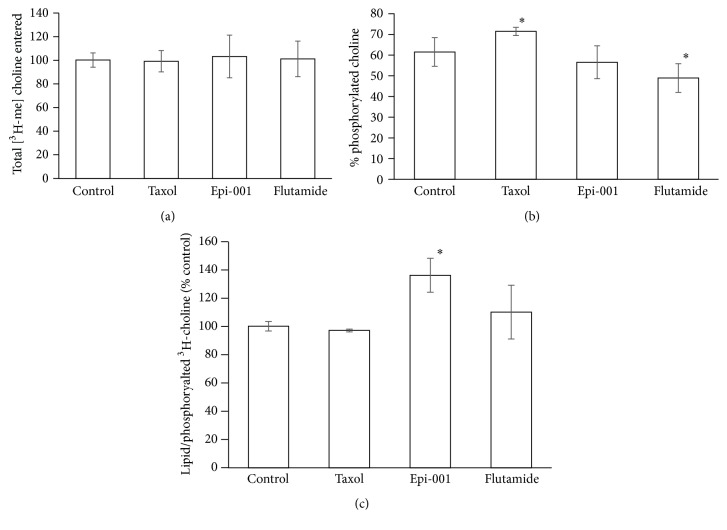
Total [^3^H-methyl]choline uptake (a), phosphorylated [^3^H-methyl]choline (as a % of total uptake) (b), and incorporation of [^3^H-methyl]choline into lipid relative to phosphorylated [^3^H-methyl]choline (c) (*∗* indicates statistically significant change compared with control).

**Figure 3:**
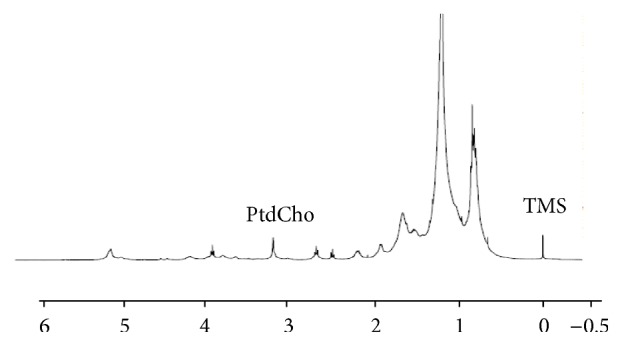
^1^H-NMR spectrum acquired from the lipid extract prepared from control LNCaP cells showing location of PtdCho resonance and the standard TMS (*x*-axis: ppm).

**Figure 4:**
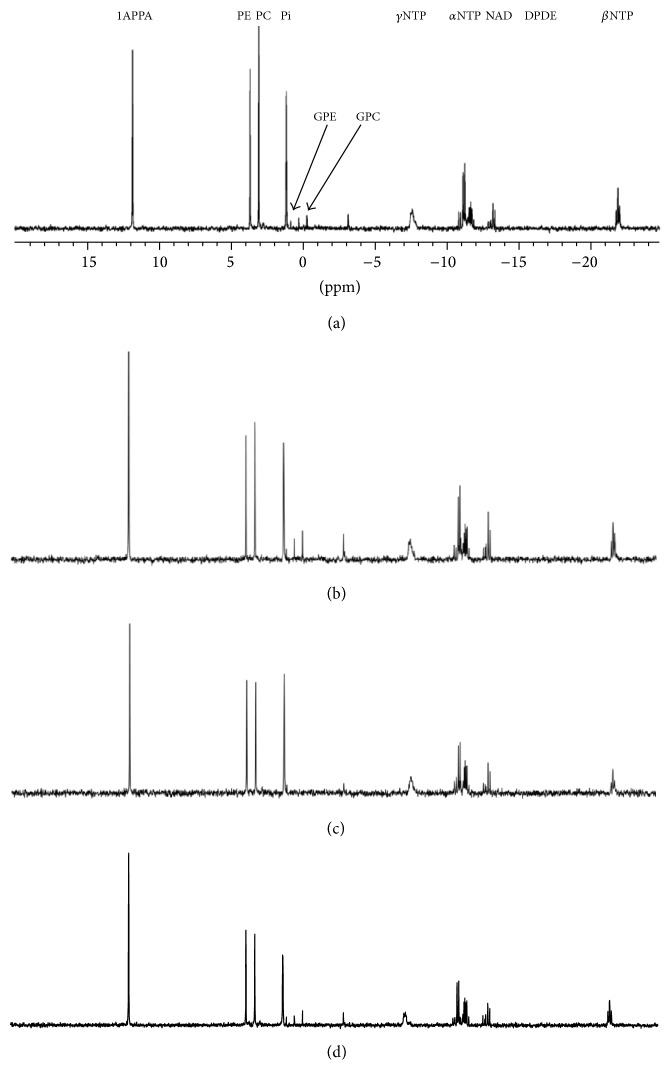
^31^P-NMR spectra acquired from chemical extracts (aqueous) prepared from untreated LNCaP cells (a) or LNCaP cells treated for 48 h with paclitaxel (5 nM) (b), Epi-1 (40 *μ*M) (c), or flutamide (20 *μ*M) (d). (1APPA: standard; PE: phosphoethanolamine; PC: phosphocholine; Pi: inorganic phosphate; GPE: glycerophosphorylethanolamine; GPC: glycerophosphorylcholine; NTP: nucleoside triphosphates; NAD: nucleotide adenine dinucleotide; DPDE: diphosphodiester sugars).

Total [^3^H-methyl]choline uptake, percent phosphorylated, and percent phosphorylated in lipid are shown in Figure 2. Total uptake is unaffected by each drug treatment, but the percentage of phosphorylated [^3^H-methyl]choline is significantly (*p* < 0.01) decreased in cells treated with flutamide. To better understand the intracellular fate of the phosphorylated [^3^H-methyl]choline, activity in the lipid fraction was determined and found to be significantly (*p* < 0.01) increased in cells treated with Epi-1. However, steady-state PtdCho content, determined by analysis of the choline peak (Figure 3) resonating at 3.4 ppm in the ^1^H spectra acquired from the lipid phase of cell extracts, was not significantly increased in cells treated with Epi (1.12 ± 0.23 *µ*mol/mg protein) compared with control cells (0.99 ± 0.03 *µ*mol/mg protein). PtdCho transfer protein (PC-TP) is involved in preserving steady-state membrane phospholipid content in cells in response to cellular lipid efflux. PC-TP is associated with the steroidal ^31^P-NMR spectroscopy that can detect phospholipid metabolites in cells and cell extracts [15, 16] and is clinically translatable [17].  Figure 4(a) shows a ^31^P-NMR spectrum acquired on an aqueous extract from untreated LNCaP cells. The standard resonates at 11.8 ppm, the phospholipid metabolites PE (phosphoethanolamine) and PC (phosphocholine) resonate at 3.75 and 3 ppm, respectively, inorganic phosphorus resonates at 1.2 ppm, the phospholipid catabolites GPE and GPC resonate at 0.5 and −0.5 ppm, respectively, PCr resonates at −3.1, and *γ*, *α*, and *β* NTP (nucleotide triphosphates) resonate at −7.5 ppm, −11, and −22 ppm, respectively. Phosphorus in NAD (nucleotide adenine dinucleotide) resonates at −11.5 and DPDE (diphosphodiesters) at −13 ppm.

**Figure 5:**
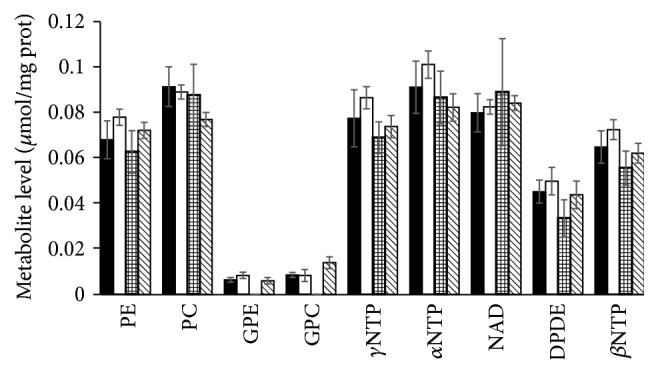
Mean (±SD)  ^31^P-NMR spectroscopy detectable metabolite content (*µ*mol/mg protein) in control (untreated (black)), and pactitaxel (5 nM) (white), Epi-1 (40 *μ*M) (squares), or flutamide (20 *μ*M) (diagonal) treated LNCaP cells.

Figure 5 shows mean (±SD) of each metabolite. In common with other studies [15], phospholipid metabolism was examined after 48 h treatments [18]. There is no significant change in the content of any of the metabolites in cells treated with either flutamide or paclitaxel compared with untreated cells. However treatment of prostate cancer cells with Epi-1 was associated with decreased levels of GPC and GPE to undetectable levels. A study [19] has shown that the level of GPC in aggressive basal-type breast tumours is greater than in the less aggressive luminal tumours. A study [20] using a inhibition of HIF1 inhibitor PX-478 which, in common with the AR-stimulated prostate cancer growth [21, 22], is associated with AMPK signalling [23] demonstrated decreased GPC in human colorectal cancer cells. A recent study [24] has demonstrated excellent resolution of PC, PE, GPC, and GPE 31P-NMR spectra in vivo from patients with prostate cancer using a 7 T system suggesting that this method could be utilised in detecting response to Epi-1.

GPC and GPE are phospholipid degradation products and have been considered measures of dynamic membrane turnover [19]. The complex role of GPC in cancer is very difficult to understand and elucidating the mechanism of therapy-induced changes in GPC is likely to be challenging [25]. GPC/E levels are the net result of several different enzyme activities including Phospholipase A2, group IV A/B, Lysophospholipase 1/2, Phospholipase B1, Phospholipase A2G3, Phospholipase A2G10, and Phospholipase A2G12A and their regulation is difficult to interpret [19]. Cellular content of GPE and GPC has recently been shown to at least in part reflect [26] the activity of the enzyme GPC-phosphodiesterase. Future work to determine the mechanism of decreased GPC and GPE could concentrate on evaluation of the expression of these enzymes in Epi-1-treated cells.

## 4. Conclusions

The protein-protein interaction inhibitor Epi-1 induces changes in phospholipid metabolism which could be exploited in monitoring response of prostate cancer to this drug using ^31^P-NMR spectroscopy.
